# Advance Care Planning Claims and Health Care Utilization Among Seriously Ill Patients Near the End of Life

**DOI:** 10.1001/jamanetworkopen.2019.14471

**Published:** 2019-11-01

**Authors:** Deepshikha Charan Ashana, Xiaoxue Chen, Abiy Agiro, Gayathri Sridhar, Ann Nguyen, John Barron, Kevin Haynes, Michael Fisch, David Debono, Scott D. Halpern, Michael O. Harhay

**Affiliations:** 1 Palliative and Advanced Illness Research Center, Perelman School of Medicine, University of Pennsylvania, Philadelphia; 2 Division of Pulmonary, Allergy, and Critical Care Medicine, Department of Medicine, Perelman School of Medicine, University of Pennsylvania, Philadelphia; 3 Leonard Davis Institute of Health Economics, University of Pennsylvania, Philadelphia; 4 Translational Research for Affordability and Quality, HealthCore Inc, Wilmington, Delaware; 5 Anthem Inc, Indianapolis, Indiana; 6 AIM Specialty Health, Deerfield, Illinois; 7 Department of Biostatistics, Epidemiology, and Informatics, Perelman School of Medicine, University of Pennsylvania, Philadelphia; 8 Department of Medical Ethics and Health Policy, Perelman School of Medicine, University of Pennsylvania, Philadelphia

## Abstract

**Importance:**

Although advance care planning is known to increase patient and caregiver satisfaction, its association with health care utilization is not well understood.

**Objective:**

To examine the association between billed advance care planning encounters and subsequent health care utilization among seriously ill patients.

**Design, Setting, and Participants:**

This retrospective cohort study conducted from October 1, 2015, to May 31, 2018, used a national commercial insurance claims database to retrieve data from 18 484 Medicare Advantage members 65 years or older who had a claim that contained a serious illness diagnosis.

**Exposure:**

A claim that contained an advance care planning billing code between October 1, 2016, and November 30, 2017.

**Main Outcomes and Measures:**

Receipt of intensive therapies, hospitalization, emergency department use, hospice use, costs, and death during the 6-month follow-up period.

**Results:**

The final study sample included 18 484 seriously ill patients (mean [SD] age, 79.7 [7.9] years; 10 033 [54.3%] female), 864 (4.7%) of whom had a billed advanced care planning encounter between October 1, 2016, and November 30, 2017. In analyses adjusted for patient characteristics and a propensity score for advance care planning, the presence of a billed advance care planning encounter was associated with a higher likelihood of hospice enrollment (incidence rate ratio [IRR], 2.52; 95% CI, 2.22-2.86) and mortality (hazard ratio, 2.27; 95% CI, 1.79-2.88) compared with no billed advance care planning encounter. Although patients with billed advance care planning encounters were also more likely to be hospitalized (IRR, 1.37; 95% CI, 1.26-1.49), including in the intensive care unit (IRR, 1.25; 95% CI, 1.08-1.45), they were less likely to receive any intensive therapies (IRR, 0.85; 95% CI, 0.78-0.92), such as chemotherapy (IRR, 0.65; 95% CI, 0.55-0.78). Similar results were observed in a propensity score–matched analysis (99% matched) and in a decedent analysis of patients who died during the 6-month follow-up period.

**Conclusions and Relevance:**

Patients with billed advance care planning encounters were more likely than those without these encounters to receive hospice services and less likely to receive any intensive therapies, such as chemotherapy. However, they were also hospitalized more frequently than patients without billed advance care planning encounters. Although these findings were robust to multiple analytic methods, the results may be attributable to residual confounding because of a higher unmeasured severity of illness in the advance care planning group. Additional evidence appears to be needed to understand the effect of advance care planning on these outcomes.

## Key Points

**Question:**

Is advance care planning associated with health care utilization among seriously ill patients?

**Findings:**

In this cohort study of 18 484 seriously ill Medicare Advantage beneficiaries, patients with billed advance care planning encounters were more likely than those without these encounters to require hospitalization, enroll in hospice, and die and less likely to receive intensive therapies, such as chemotherapy.

**Meaning:**

Among seriously ill patients near the end of life, advance care planning may not be assoicated with reduced health care utilization.

## Teaser

This cohort study examines the association between billed advance care planning encounters and subsequent health care utilization among seriously ill patients near the end of life.

## Introduction

Advance care planning (ACP) is a critical component of high-quality end-of-life care.^1,2^ Timely discussion about care preferences supports the delivery of goal-concordant care, increases patient and caregiver satisfaction, and may decrease costs through avoidance of unwanted, high-intensity interventions.^3,4,5,6,7,8,9,10^ The lack of a reliable marker for ACP in administrative claims has limited the scope of past research. Specifically, the association of ACP with health care utilization in a national sample of patients is not well understood. This is a major limitation in light of the importance of such metrics for many stakeholders, including payers.^11^ However, the recent introduction of billing codes for the provision of ACP services has the potential to facilitate observational research of ACP on a broader scale than has previously been possible. These codes were introduced by the Centers for Medicare & Medicaid Services in January 2016 to reimburse practitioners for any discussion about ACP.^12,13,14,15^

In this context, the goal of our study was to examine whether having a billed ACP encounter is associated with subsequent health care utilization among seriously ill patients. We hypothesized that seriously ill patients with billed ACP encounters would have higher rates of hospice enrollment and mortality, lower rates of hospitalization and intensive therapy (such as intubation) use, and lower costs than patients without such encounters.

## Methods

### Data Source

This retrospective cohort study used administrative medical and pharmacy claims data from the HealthCore Integrated Research Database (HIRD). The HIRD is a repository of fully adjudicated claims data from 14 commercial health plans across the United States. A prior study^16^ compared 14.8 million HIRD enrollees with 307.7 million individuals from the 2009 US Census and found that the HIRD database may underrepresent individuals older than 65 years and those who live in the southern United States and overrepresent those who live in the midwestern United States. This database was chosen because Medicare Advantage beneficiaries are not often included in epidemiologic studies despite representing a large and increasing proportion of Medicare beneficiaries, estimated at 34% in 2018.^17^ This study was conducted in full compliance with relevant provisions of the Health Insurance Portability and Accountability Act (HIPAA).^18^ The researchers performed secondary data analysis on administrative claims data. The researchers only had access to an analytic file derived from a limited data set with a data use agreement that specifies rules of using data for research and health plan operation purposes (as defined by the HIPAA Standards for Privacy of Individually Identifiable Health Information [Privacy Rule]^19^). Because the researchers worked on an analytic file that does not contain date of birth, date of service, address, and contact information to perform analysis, no waiver of informed consent approval or exemption was needed from an institutional review board per 45 §CFR 164.514(e). This study follows the Strengthening the Reporting of Observational Studies in Epidemiology (STROBE) reporting guideline.

### Study Population

The study population included Medicare Advantage members 65 years or older who had a claim that contained an *International Statistical Classification of Diseases and Related Health Problems, Tenth Revision (ICD-10)* diagnosis code for serious illness between October 1, 2015, and September 30, 2016. The *ICD-10* diagnosis codes for serious illness have been used as inclusion criteria in several randomized clinical trials.^20,21^ The serious illness algorithm identified patients with chronic obstructive pulmonary disease, Alzheimer disease and related dementias, fibrotic lung disease, advanced solid malignant tumors, neurodegenerative conditions, renal disease, or heart failure who were expected to have a median survival of less than 2 years or significant debility as a result of their disease.

**Figure 1.**
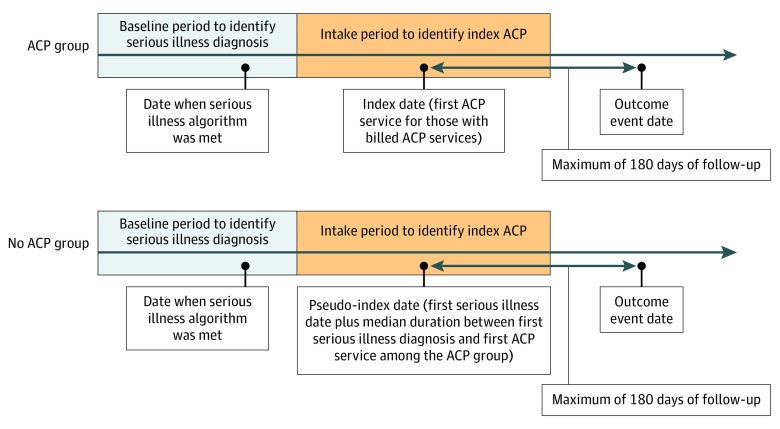
Patient Cohort Specification All patients had a claim that contained a diagnosis code for a serious illness during the baseline period of October 1, 2015, to September 30, 2016. Patients with a billed advanced care planning (ACP) encounter during the intake period of October 1, 2016, to November 30, 2017, were included in the ACP group, whereas patients without a billed ACP encounter during the intake period were included in the no ACP group. The earliest date of ACP code use was assigned as the index date for patients in the ACP group. The pseudo-index date for patients in the no ACP group was calculated by adding an offset after their first serious illness diagnosis date that was derived from the distribution of days between the first serious illness diagnosis and first ACP code use in the ACP group. Outcomes were assessed in the 180 days after the index or pseudo-index date.

The ACP group included patients with at least 1 billed ACP encounter, defined as the presence of *Current Procedural Terminology* codes 99497 or 99498 between October 1, 2016, and November 30, 2017. Patients who had an ACP encounter before October 1, 2016, were excluded from the study. The earliest date of ACP code use was assigned as the index date. Patients without billed ACP encounters were included in the comparison group (no ACP group). The pseudo-index date for these patients was calculated by adding an offset after the date of their first serious illness diagnosis that was derived from the distribution of days between the first serious illness diagnosis and first ACP code use in the ACP group (Figure 1). Continuous medical and pharmacy enrollment was required for 1 full year before the index or pseudo-index date.

### Outcome Measures

Clinical, health care utilization, and cost outcomes were measured within the 6-month follow-up period after the index or pseudo-index date (Figure 1). Follow-up time began after the index or pseudo-index date and ended at a censoring event (death), end of the follow-up period (6 months after the index or pseudo-index date), or end of the study period (May 31, 2018), whichever came first. The clinical outcome was death as ascertained from the Social Security Death Master File,^22,23^ hospital claim discharge status records, or health plan disenrollment records. Health care utilization outcomes included the following intensive therapies: intubation and mechanical ventilation, tracheostomy, gastrostomy tube insertion, dialysis, enteral or parenteral nutrition, and chemotherapy.^24^ Health care utilization outcomes also included hospitalization with and without intensive care unit (ICU) admission, emergency department (ED) visits, and receipt of hospice services.^25^ Cost outcomes included costs of total medical care, including hospitalization, ED visits, skilled nursing facility care, and all forms of outpatient care, including hospice care, physician visits, and home health care, as well as pharmacy services. Cost outcomes were defined as the allowed amount, which captures both plan-paid and patient-paid amounts. All spending was adjusted to 2017 values using the Consumer Price Index.

### Statistical Analysis

Descriptive statistics, including means (SDs) for continuous data and numbers (percentages) for categorical data, were measured. Differences in the descriptive characteristics between the comparison groups during the baseline period were assessed using standardized differences. All analyses were conducted using SAS Enterprise Guide, version 7.1 (SAS Institute Inc).

Regression modeling with adjustment for selected covariates or use of the propensity score^26^ (ie, the probability of a billed ACP encounter) are 2 strategies to reduce confounding in effect estimates in observational studies. Both have potential advantages and may also be used together, which is known as doubly robust estimation.^27^ The motivation to use the doubly robust approach is that it combines these approaches such that only 1 of the models must be correctly specified. We considered the same set of covariates as those considered for traditional covariate adjustment in the regression models to calculate the propensity score. These covariates were demographic variables (age, sex, and region), Deyo-Charlson Comorbidity Index,^28^ behavioral health conditions (depression, anxiety, and adjustment disorder), and baseline all-cause medical and hospitalization costs. Before assessing outcomes, the validity of the propensity score modeling was assessed by graphically examining the overlap in propensity scores between groups (eFigure in the Supplement). In addition to the doubly robust analysis, we conducted a propensity score–matched analysis by 1:3 pairing of a patient in the ACP group with patients in the no ACP group based on their propensity scores using a greedy matching algorithm.^29^ We retained 855 of 864 total patients (99.0%) in the ACP group after matching. We compared the balance in baseline patient characteristics after propensity score matching using standardized differences (eTable 1 in the Supplement).

Generalized estimating equation regression with log link and Poisson distribution were used to compare rates of health care outcomes between the ACP and no ACP groups. Cox proportional hazards regression was used to compare the mortality rate between the ACP and no ACP groups. For cost outcomes, per-patient per-month (PPPM) differences between the 2 groups were reported to account for varying lengths of follow-up. Generalized estimating equation regression with log link and γ distribution were used to compare PPPM cost differences between the 2 groups.

Because the foregoing methods may not fully adjust for confounding because of differences in severity of illness between the 2 groups, we additionally tested the robustness of our results by conducting an analysis restricted to patients who died during the 6-month follow-up period. The logic behind a decedent analysis is that the sample of included patients may have less residual confounding because of severity of illness because all patients died and thus represent the sickest individuals.

## Results

### Unadjusted Patient Comparisons

The final study sample included 18 484 seriously ill patients (mean [SD] age, 79.7 [7.9] years; 10 033 [54.3%] female), 864 (4.7%) of whom had a billed advanced care planning encounter between October 1, 2016, and November 30, 2017. Among those with an ACP encounter, the mean (SD) number of ACP encounters per patient was 1.2 (0.5), with a maximum of 4 ACP encounters. Most ACP services were provided by primary care physicians (355 [41.1%]) and nonphysician practitioners (179 [20.7%]), such as nurse practitioners, in outpatient care settings (770 [89.1%]). Compared with the 17 620 patients without a billed ACP encounter, those with ACP claims were older (mean [SD], 81.4 [8.2] vs 79.6 [7.8] years), had more comorbid illnesses based on the Deyo-Charlson Comorbidity Index (mean [SD], 6.0 [3.1] vs 4.6 [2.8]), and had similar numbers of serious illness diagnoses (mean [SD, 1.4 [0.8] vs 1.3 [0.7]). Patients with ACP claims had greater health care utilization in the previous year than patients without ACP claims, with more hospital admissions (mean [SD], 2.0 [2.1] vs 0.9 [1.5]) and ED visits (mean [SD], 1.0 [1.6] vs 0.8 [1.5]) during the preceding year and commensurately higher median total medical costs ($31 044 [interquartile range {IQR}, $10 103-$68 754] vs $9565 [IQR, $3218-$28 546]) (Table 1).

**Table 1.** Baseline Characteristics of Seriously Ill Patients With and Without Billed Advance Care Planning Encounters^a^

Characteristic	All Patients With Serious Illness			Patient Decedents		
	With ACP Claim (n = 864)	Without ACP Claim (n = 17 620)	Standardized Difference^b^^,^^c^	With ACP Claim (n = 86)	Without ACP Claim (n = 520)	Standardized Difference^b^^,^^d^
Age, mean (SD), y	81.4 (8.2)	79.6 (7.8)	0.22	83.1 (7.5)	81.5 (8.1)	0.21
Sex			–0.10			–0.21
Male	394 (45.6)	8057 (45.7)	–0.01	45 (52.3)	270 (51.9)	0.01
Female	470 (54.4)	9563 (54.3)	0.01	41 (47.7)	250 (48.1)	–0.01
Region			0.33			0.46
Northeast	233 (27.0)	3287 (18.7)	0.20	44 (51.2)	155 (29.8)	0.45
Midwest	439 (50.8)	11 756 (66.7)	–0.33	28 (32.6)	274 (52.7)	–0.42
South	167 (19.3)	2160 (12.3)	0.19	10 (11.6)	69 (13.3)	–0.05
West	24 (2.8)	393 (2.2)	0.04	4 (4.7)	20 (3.8)	0.04
Deyo-Charlson Comorbidity Index, mean (SD)	6.0 (3.1)	4.6 (2.8)	0.47	7.5 (3.2)	6.1 (3.1)	0.43
No. of Deyo-Charlson comorbidities			0.27			0.29
0	6 (0.7)	569 (3.2)	–0.18	0	7 (1.3)	–0.17
1-2	30 (3.5)	1346 (7.6)	–0.18	0	14 (2.7)	–0.24
≥3	828 (95.8)	15 705 (89.1)	0.26	86 (100)	499 (96.0)	0.29
No. of serious illness diagnoses, mean (SD)	1.4 (0.8)	1.3 (0.7)	0.12	1.5 (0.8)	1.6 (0.9)	–0.09
Serious illness diagnosis						
COPD	198 (22.9)	3572 (20.3)	0.06	22 (25.6)	139 (26.7)	–0.03
ADRD	127 (14.7)	1946 (11.0)	0.11	10 (11.6)	63 (12.1)	–0.02
Fibrotic lung disease	76 (8.8)	1434 (8.1)	0.02	11 (12.8)	64 (12.3)	0.01
Advanced solid malignant neoplasm	108 (12.5)	2652 (15.1)	–0.07	10 (11.6)	95 (18.3)	–0.19
Neurodegenerative disease	1 (0.1)	92 (0.5)	–0.07	0	3 (0.6)	–0.11
ESRD	21 (2.4)	273 (1.5)	0.06	3 (3.5)	27 (5.2)	–0.08
Heart failure	696 (80.6)	13 482 (76.5)	0.10	74 (86.0)	433 (83.3)	0.08
No. of hospital admissions, mean (SD)	2.0 (2.1)	0.9 (1.5)	0.62	3.0 (1.8)	1.8 (1.9)	0.66
No. of ED visits, mean (SD)	1.0 (1.6)	0.8 (1.5)	0.17	1.1 (1.6)	1.0 (1.5)	0.10
Total medical costs, median (IQR), $^e^	31 044 (10 103-68 754)	9565 (3218-28 546)	0.58	67 832 (35 503-93 535)	27 312 (10 135-65 769)	0.54
Hospitalization	17 626 (0-44 887)	0 (0-12 914)	0.61	45 311 (21 988-72 891)	11 750 (0-33 056)	0.68
Outpatient services	7022 (3365-14 179)	4831 (2301-9963)	0.16	9857 (5435-16 245)	7775 (3630-18 424)	–0.14
ED	0 (0-1808)	0 (0-983)	0.18	524 (0-2664)	0 (0-1390)	0.24
Total pharmacy costs, median (IQR), $	2922 (1003-6392)	2482 (889-5592)	0.03	3734 (1298-8399)	3494 (1261-7131)	0.01

Abbreviations: ACP, advance care planning; ADRD, Alzheimer disease and related dementias; COPD, chronic obstructive pulmonary disease; ED, emergency department; ESRD, end-stage renal disease; IQR, interquartile range.

^a^ Data are presented as number (percentage) of participants unless otherwise indicated.

^b^ The standardized difference is the number of SDs that separate the 2 groups. An absolute value greater than 0.2 (ie, 20% of an SD) represents a meaningful difference.

^c^ Comparing all patients with an ACP claim and all patients without an ACP claim.

^d^ Comparing decedents with an ACP claim and all decedents without an ACP claim.

^e^ Includes costs paid by the health plan and the patient.

Compared with the 18 484 patients in the study sample, the 606 patients who died during the 6-month follow-up period were older and had more comorbidities, hospital admissions, and medical expenditures. Decedents were also more likely than all seriously ill patients to have a billed ACP encounter (86 [14.2%] vs 864 [4.7%]). Results similar to those described above were observed when comparing the 86 decedents who had an ACP claim with the 520 decedents who did not have an ACP claim (Table 1).

### Adjusted Patient Comparisons

**Figure 2.**
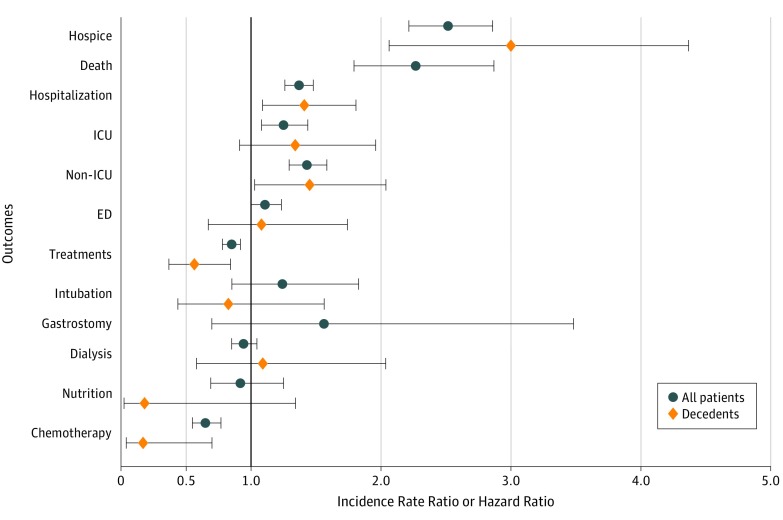
Doubly Robust Analysis of Outcomes of Seriously Ill Patients With vs Without a Billed Advance Care Planning Encounter A propensity score and all covariates listed in Table 1 were included in the regression analysis. Outcomes were measured during the 180-day follow-up period and included hospice enrollment; death; any hospitalization, including hospitalization with intensive care unit (ICU) admission and hospitalization without ICU admission; emergency department (ED) visit; and receipt of any intensive life support therapy, including intubation, gastrostomy tube placement, dialysis, artificial nutrition, and chemotherapy. Incidence rate ratios were not calculated for gastrostomy tube placement or death among decedents because no patients in the advance care planning group had a gastrostomy tube placed and all decedents in both groups died during the follow-up period. For all outcomes, incidence rate ratios were estimated, with the exception of mortality, for which a hazard ratio was estimated. Error bars indicate 95% CIs.

In doubly robust analyses adjusted for patient characteristics and the propensity score, having a billed ACP encounter was associated with higher rates of hospice enrollment (incidence rate ratio [IRR], 2.52; 95% CI, 2.22-2.86) and mortality (hazard ratio, 2.27; 95% CI, 1.79-2.88) (Figure 2). However, the time to first hospice enrollment was similar between groups (median, 53 days; IQR, 11-86 days for the ACP group vs median, 53 days; IQR, 18-110 days for the no ACP group). Patients with a billed ACP encounter were also more likely to be hospitalized (IRR, 1.37; 95% CI, 1.26-1.49), including hospitalizations with (IRR, 1.25; 95% CI, 1.08-1.45) and without (IRR, 1.43; 95% CI, 1.29-1.58) ICU admission. However, they did not have a significantly higher incidence of ED visits (IRR, 1.11; 95% CI, 0.99-1.24). Patients with ACP claims were less likely to receive intensive therapies (IRR, 0.85; 95% CI, 0.78-0.92), most notably chemotherapy (IRR, 0.65; 95% CI, 0.55-0.78). In addition, among patients who were receiving chemotherapy in the 12 months before the index or pseudo-index date, those with an ACP claim were more likely to discontinue chemotherapy during the follow-up period than those without an ACP claim (IRR, 0.63; 95% CI, 0.52-0.78). Similar associations were observed within the decedent subgroup (Figure 2).

Total medical costs were higher among patients with a billed ACP encounter than among other patients (mean adjusted PPPM cost difference, $1635; 95% CI, $1243-$2075), a difference that was largely associated with hospital costs (mean adjusted PPPM cost difference, $1443; 95% CI, $891-$2142). Total medical costs were also higher among decedents than among all patients with serious illnesses, with the highest costs observed among decedents who had an ACP claim (Table 2). Results of the propensity score–matched analysis were consistent with the main findings reported here (eTable 2 and eTable 3 in the Supplement).

**Table 2.** Medical and Pharmacy Costs for Seriously Ill Patients With and Without Billed ACP Encounters in Doubly Robust Analyses^a^

Cost	All Patients With Serious Illness				Patient Decedents			
	With ACP Claim	Without ACP Claim	Mean Adjusted Cost Difference (95% CI)	*P* Value	With ACP Claim	Without ACP Claim	Mean Adjusted Cost Difference (95% CI)	*P* Value
Total PPPM medical costs, median (IQR), $	1401 (266 to 5210)	484 (155 to 2257)	1635 (1243 to 2075)	<.001	7622 (0 to 20 119)	8625 (3216 to 17 115)	15 835 (3041 to 38 069)	.01
Hospitalization	0 (0 to 2556)	0 (0 to 522)	1443 (891 to 2142)	<.001	207 (0 to 16 016)	6287 (933 to 13 822)	15 798 (886 to 47 393)	.03
ED	0 (0 to 12)	0 (0 to 0)	–1 (–13 to 13)	.87	0 (0 to 0)	0 (0 to 204)	–239 (–284 to –126)	.01
Pharmacy PPPM costs, median (IQR), $	168 (42 to 475)	188 (64 to 484)	–77 (–114 to –37)	<.001	29 (0 to 238)	178 (44 to 547)	–157 (–352 to 212)	.32

Abbreviations: ACP, advance care planning; ED, emergency department; IQR, interquartile range; PPPM, per-patient per-month.

^a^All US dollar amounts were adjusted to 2017 values using the Consumer Price Index.

## Discussion

In a national sample of seriously ill patients with Medicare Advantage insurance coverage, having a billed ACP encounter was associated with a higher likelihood of hospice enrollment and mortality in the 6 months after the index ACP visit. Although these patients were also more likely to be hospitalized, including in the ICU, they were less likely to receive intensive therapies. We are unable to make inferences about rates of specific therapies because of low overall event rates for all therapies other than chemotherapy and dialysis. In addition, patients with ACP encounters accrued greater total health care costs that were primarily associated with their hospitalizations.

The finding that patients with billed ACP encounters were more likely to use hospice services may suggest that these patients preferred palliative rather than restorative care. However, the effect of the billed ACP encounter on the eliciting or shaping of these preferences cannot be discerned by our study. Such care preferences may also explain the observed increase in mortality among these patients, a hypothesis that is supported by their lower likelihood of receiving intensive therapies. Another potential explanation for the difference in hospice use may be that patients with billed ACP encounters were sicker than those without such encounters, and our primary analysis failed to fully control for these differences. This finding was robust in a decedent subgroup analysis that was expected to reduce but not eliminate the possibility of residual confounding.

Although patients with billed ACP encounters were more likely to be hospitalized and admitted to the ICU, also possibly because of uncontrolled differences in severity of illness, they were less likely to receive intensive therapies. These seemingly inconsistent results might be reconciled if the care received during hospital stays was different for patients with and without ACP claims. For example, seriously ill patients who are hospitalized for symptom management or further clarification of goals of care during an acute deterioration in their health may not receive the intensive therapies that would otherwise be medically indicated. If this hypothesis is confirmed in future studies, it would suggest that end-of-life hospital and ICU utilization may be patient centered in some cases.^30^

### Strengths and Limitations

This study has several strengths. To our knowledge, these data were among the first to use ACP billing codes to examine the association of ACP with patient outcomes. In addition, this study included a national sample of seriously ill patients and used a unique database of health claims that includes a diverse group of patients with Medicare Advantage coverage.

Our study also has several limitations. First, there is a probability of exposure misclassification. Specifically, the absence of an ACP claim does not preclude an ACP conversation, and the presence of a claim may not be associated with the quality or comprehensiveness of ACP. In addition, patients who had previously engaged in ACP or documented their care wishes may have been less likely to have a billed ACP encounter. However, such misclassification would tend to bias our results toward the null, suggesting that the associations that we observed may be underestimates. Second, in the absence of clinical data for severity adjustment, including actual and perceived severity of illness, it is likely that our results were partially influenced by residual patient-level confounding. To mitigate this concern, we selected patients with a high severity of illness using serious illness inclusion criteria and performed both propensity score–matched and decedent analyses. However, statistical methods are unlikely to fully eliminate this bias. Third, observational studies of ACP are prone to confounding by indication because patients’ preexisting preferences may influence the likelihood of ACP conversations and end-of-life health care utilization. Despite this limitation, observational studies with more generalizable populations, such as ours, are an efficient adjunct to the randomized clinical trials that are ultimately needed to build a robust evidence base for end-of-life care interventions.^31^ In addition, once use of the ACP billing codes is more prevalent, future observational studies should consider using practitioners’ billing preferences as an instrumental variable to facilitate causal inferences about the effect of ACP on health care utilization.^32^ Fourth, the findings of this study may not be generalizable to patients enrolled in Medicare fee-for-service plans, who have previously been found to be sicker and more likely to use health care resources than patients enrolled in Medicare Advantage plans.^33,34^ Fifth, our database did not include important patient covariates, such as patient race/ethnicity or marital status, and practitioner covariates, such as health care utilization rates. Sixth, it is possible that our approach to deriving the pseudo-index date influenced our reported variance estimates. However, there are no clearly superior alternative approaches, and any introduced bias or imprecision is likely to be small compared with the size of the effect estimates.^35^

## Conclusions

In this national sample of seriously ill patients, patients with billed ACP encounters had higher likelihoods of mortality and hospice enrollment and a lower likelihood of receiving life-sustaining therapies. Future randomized clinical trials appear to be necessary to further evaluate the causal effect of ACP on these patient outcomes.

## Notes

Supplement. **eFigure.** Overlap in Propensity Score Between Patients With and Without Billed Advance Care Planning Encounters **eTable 1.** Characteristics of Seriously Ill Patients With and Without Billed Advance Care Planning Encounters After 1:3 Propensity Score Matching **eTable 2.** Propensity Score Matched Analysis of Outcomes of Seriously Ill Patients With a Billed Advance Care Planning Encounter Compared With Patients Without a Billed Advance Care Planning Encounter **eTable 3.** Medical and Pharmacy Costs for Seriously Ill Patients With and Without Billed Advance Care Planning Encounters in 1:3 Propensity Score Matched Analyses
