# Supplementary material for: Advance Care Planning Claims and Health Care Utilization Among Seriously Ill Patients Near the End of Life
Source: JAMA Netw Open. 2019 Nov 1;2(11):e1914471. doi: 10.1001/jamanetworkopen.2019.14471 (PMC6827391; doi:10.1001/jamanetworkopen.2019.14471)
Supplement: Supplement. — eFigure. Overlap in Propensity Score Between Patients With and Without Billed Advance Care Planning Encounters eTable 1. Characteristics of Seriously Ill Patients With and Without Billed Advance Care Planning Encounters After 1:3 Propensity Score Matching eTable 2. Propensity Score Matched Analysis of Outcomes of Seriously Ill Patients With a Billed Advance Care Planning Encounter Compared With Patients Without a Billed Advance Care Planning Encounter eTable 3. Medical and Pharmacy Costs for Seriously Ill Patients With and Without Billed Advance Care Planning Encounters in 1:3 Propensity Score Matched Analyses [file jamanetwopen-2-e1914471-s001.pdf]

Supplementary Online Content

Ashana DC, Chen X, Agiro A, et al. Advance care planning claims and health care utilization among seriously ill patients near the end of life. *JAMA Netw Open*. 2019;2(11):e1914471. doi:10.1001/jamanetworkopen.2019.14471

eFigure. Overlap in Propensity Score Between Patients With and Without Billed Advance Care Planning Encounters

eTable 1. Characteristics of Seriously Ill Patients With and Without Billed Advance Care Planning Encounters After 1:3 Propensity Score Matching

eTable 2. Propensity Score Matched Analysis of Outcomes of Seriously Ill Patients With a Billed Advance Care Planning Encounter Compared With Patients Without a Billed Advance Care Planning Encounter

eTable 3. Medical and Pharmacy Costs for Seriously Ill Patients With and Without Billed Advance Care Planning Encounters in 1:3 Propensity Score Matched Analyses

This supplementary material has been provided by the authors to give readers additional information about their work.

© 2019 Ashana DC et al. *JAMA Network Open.*

eFigure. Overlap in propensity score between patients with and without billed advance care planning encounters.

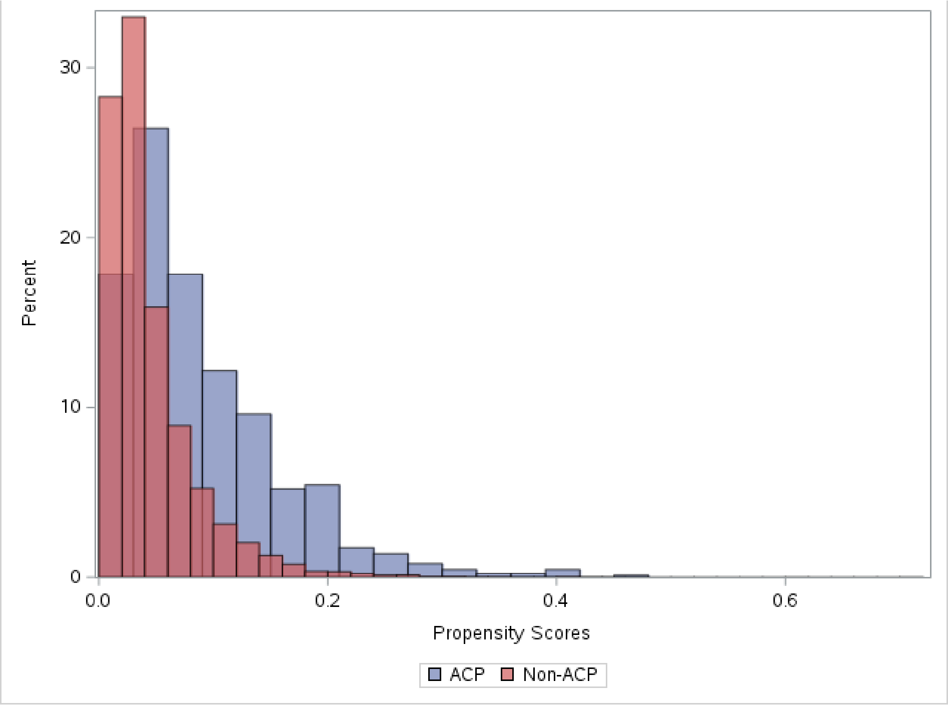

Definition of abbreviations: ACP = advance care planning. The likelihood of having a billed ACP encounter was modeled as a logistic regression adjusting for demographic variables (age, female gender and census region), Deyo-Charlson Comorbidity Index, behavioral health conditions (depression, anxiety, adjustment disorder), and baseline all-cause medical and hospitalization costs. The propensity scores indicate the probability of having a billed ACP encounter and range from 0 to 1.

© 2019 Ashana DC et al. *JAMA Network Open.*

eTable 1. Characteristics of seriously ill patients with and without billed advance care planning encounters after 1:3 propensity score matching

Patient characteristics	All patients with serious illness		
	**Patients receiving ACP[a]**	**Patients without ACP claim**	**Standardized difference [b]**
Patients, No.	855	2,565	
Age, mean (SD)	81.4 (8.2)	81.5 (7.9)	-0.02
Gender, %			0.03
Male	45.7	46.0	-0.01
Female	54.3	54.0	0.01
Region, %			0.02
Northeast	27.0	26.2	0.02
Midwest	51.1	51.6	-0.01
South	19.1	19.2	-0.01
West	2.8	3.1	-0.02
Dey-Charlson Comorbidity Index, mean (SD)	6.0 (3.1)	5.9 (3.2)	0.02
Number of Deyo-Charlson Comorbidities, %			0.00
0	0.7	1.3	-0.06
1-2	3.5	3.9	-0.02
≥3	95.8	94.9	0.04
Number of serious illness diagnoses, mean (SD)	1.4 (0.8)	1.4 (0.8)	-0.01
Serious illness diagnosis, %			
COPD	22.9	21.1	0.05
ADRD	14.7	13.7	0.03
Fibrotic lung disease	8.8	8.9	-0.01
Advanced solid malignancy	12.4	16.0	-0.10
Neurodegenerative disease	0.1	0.5	-0.07
ESRD	2.3	2.4	-0.01
Heart failure	80.4	80.2	0.01
Number of hospital admissions, mean (SD)	2.0 (2.0)	1.9 (2.2)	0.01
Number of ER visits, mean (SD)	1.0 (1.5)	1.1 (2.2)	-0.06
Total medical costs, mean $ (SD) [c]	45,691 (46,512)	43,584 (49,659)	0.04
Hospitalization	29,841 (37,832)	26,329 (39,716	0.09
Outpatient services	12,401 (16,225)	14,012 (19,570)	-0.09
ER	1,227 (2,075)	1,292 (2,494)	-0.03
Total pharmacy costs, mean $ (SD)	5,998 (12,440)	5,851 (12,518)	0.01

Definition of abbreviations: ACP = advance care planning; ADRD = Alzheimer's disease and related dementias; COPD = chronic obstructive pulmonary disease; ER = emergency room; ESRD = end-stage renal disease; SD = standard deviation.

[a] We retained 855 of 864 total patients (99.0%) in the ACP group after matching.
[b] Comparing all patients receiving ACP and all patients without an ACP claim.
[c] Includes costs paid by the health plan and the patient.

© 2019 Ashana DC et al. *JAMA Network Open.*

eTable 2. Propensity score matched analysis of outcomes of seriously ill patients with a billed advance care planning encounter compared to patients without a billed advance care planning encounter

Outcomes	IRR	95% CI	P value
**Hospice use**	2.38	2.06-2.76	<0.001
**Death**	2.03	1.55-2.65	<0.001
**Hospitalization**	1.27	1.16 - 1.39	<0.001
**With ICU admission**	1.16	0.98 - 1.36	0.08
**Without ICU admission**	1.33	1.19 - 1.49	<0.001
**Emergency room visit**	1.07	0.95 - 1.20	0.29
**Intensive therapy use**	0.74	0.68 - 0.81	<0.001
**Intubation and mechanical ventilation**	1.26	0.82 - 1.93	0.29
**Gastrostomy tube insertion**	1.28	0.54 - 3.05	0.58
**Dialysis**	0.74	0.66 - 0.82	<0.001
**Enteral or parenteral nutrition**	1.06	0.77 - 1.47	0.71
**Chemotherapy**	0.62	0.51 - 0.74	<0.001

Definition of abbreviations: CI = confidence interval; IRR = incidence rate ratio.

© 2019 Ashana DC et al. *JAMA Network Open.*

eTable 3. Medical and pharmacy costs for seriously ill patients with and without billed advance care planning encounters in 1:3 propensity score matched analyses

Costs	Patients receiving ACP [a]	Patients without ACP claim	Cost difference	P value
Total PMPM medical costs, mean $ (SD)	10,094 (132,603)	3,586 (13,974)	6,508	<0.001
Hospitalization	8,564 (132,631)	2,326 (13,696)	6,238	0.83
ER	117 (394)	128 (655)	-10	0.92
Pharmacy PMPM costs, mean $ (SD)	423 (1,117)	491 (1,207)	-68	0.02

Definition of abbreviations: ER = emergency room; PMPM = per member per month; SD = standard deviation. All US dollar amounts were adjusted to 2017 values using the consumer price index.
[a] We retained 855 of 864 total patients (99.0%) in the ACP group after matching.

© 2019 Ashana DC et al. *JAMA Network Open.*
